# Shape-similarity gain: A selection profile for simple objects

**DOI:** 10.3758/s13414-026-03283-y

**Published:** 2026-06-09

**Authors:** Brendan Valentine, Xiaoli Zhang, Taosheng Liu

**Affiliations:** Department of Psychology, Michigan State University, 316 Physics Rd, East Lansing, MI 48824 USA

**Keywords:** Attention: Selective, Similarity, Attention: object-based

## Abstract

Recent research has examined the selection profile—the pattern of enhancement and suppression of stimuli when allocating attention to a specific feature value. Support for several different selection profiles for simple feature dimensions (e.g., color, orientation) have been identified such as the feature-similarity gain and surround suppression effects. However, it is unclear whether the selection profile for more complex stimuli follows a similar pattern. Using a recently developed circular shape space, the current study assessed the selection profile for these simple shapes as an initial step toward the study of object-based attention. Participants attended to two target shapes while ignoring two distractors, each overlapping with one target. They performed a change identification task, reporting luminance changes of only the target shapes. We systematically varied the difference between the two targets in the shape space. In two experiments covering different ranges in the shape space, performance accuracy peaked when the two target shapes matched and then decreased in a monotonic manner as the two shapes became more different. These results align with a feature-similarity gain model and suggest that an analogous shape-similarity gain effect operates for more complex stimuli. This gain effect suggests that shape-based selection occurs in an intrinsic similarity space which can be realized by top-down attention operating on neural substrates that encode object similarity.

**Supplementary Information:**

The online version contains supplementary material available at 10.3758/s13414-026-03283-y.

Selective attention allows us to pick out relevant information from the vast stream of sensory stimuli that bombard our perceptual systems every day. From simply scanning the refrigerator for a snack to ignoring unimportant landscapes and billboards while driving in stormy weather, the function of visual selective attention is vital. Decades of research has shown that attention to both spatial location and simple features enhance processing of potential targets matching the attended location or feature, making these matching stimuli more likely to be selected for prioritized processing (Carrasco, 2011; Liu, 2019; Maunsell & Treue, 2006; Scolari et al., 2014). Here, we refer to the pattern of potential enhancement and suppression of stimuli when attending to a piece of key information as the *selection profile* of visual attention. In the domain of feature-based attention, this selection profile has been widely studied, and two main models have emerged in the literature.

The dominant model for the selection profile of feature-based attention has been the feature-similarity gain model, originally proposed based on neurophysiological studies in nonhuman primates (Martinez-Trujillo & Treue, 2004; Treue & Martinez-Trujillo, 1999). According to the model, when we attend to a feature (e.g., the color red), attention enhances all red-colored items in the scene and such enhancement gradually declines as an item’s color becomes less reddish, to the point that the most dissimilar features are suppressed (Martinez-Trujillo & Treue, 2004). In addition to evidence from nonhuman primates, this model has also received support from human psychophysical and neuroimaging studies (Bondarenko et al., 2012; Boynton et al., 2006; Liu et al., 2007; Serences & Boynton, 2007; Wang et al., 2015; Zhang & Luck, 2008). Overall, this evidence suggests a mechanism that produces a monotonic selection profile through a combination of enhancement of the target feature and suppression of the most different features within the same dimension.

In contrast to the monotonic profile proposed by the feature-similarity gain model, more recent studies have found a nonmonotonic pattern of the selection profile where maximum suppression occurs for nearby, competitive distractors, with a rebound in performance beyond this point. This pattern has been referred to as the *surround suppression* profile, with evidence coming from both behavioral and neuroimaging studies (Bartsch et al., 2017; Fang & Liu, 2019; Fang et al., 2019; Ho et al., 2012; Liu et al., 2023; Störmer & Alvarez, 2014; Tombu & Tsotsos, 2008; Yoo et al., 2018). While the precise condition that elicits either a monotonic or a nonmonotonic selection profile is still unknown, recent work suggests that certain task factors could play a role. For example, while similarity gain seems to be active across tasks of varying complexity, surround suppression is only engaged when it is adaptive, specifically when there is a high level of stimulus competition, or a need for precise selection (Liu et al., 2023). This promotes the idea that similarity gain is a general mechanism for selecting targets from distractors, while surround suppression is a more context-dependent mechanism that is engaged only under challenging circumstances.

Thus far, these selection profiles have only been observed when attention is guided to a specific low-level feature; however, the natural environment is made up of more complex stimuli than tilted bars, moving dots, and red patches. While features are often useful attentional guides for real-world visual tasks, objects are almost always the unit of attentional selection (Scholl, 2001). Features are often the descriptors of the actual item we are searching for, where in “red dots” the feature “red” describes an aspect of the object “dot.” Given this reliance on objects, our understanding of the selection mechanism would benefit from examining how attention operates on object representations. However, measuring the selection profile for object-based attention is complicated by the fact that objects reside in a high-dimensional space that does not have a simple geometry like color or orientation space. Although recent data-driven approaches have shown that complex natural objects can be represented in low-dimensional spaces (Bao et al., 2020; DiCarlo et al., 2012; Hebart et al., 2020; Yao et al., 2023; Younes, 2012), the psychological relevance of such spaces is still unclear. An alternative, and perhaps more intuitive, approach is to leverage subjective similarity to directly construct such a space. For example, Li et al. (2020) created a “shape wheel” analogous to a wheel in a standard color space (Fig. 1). This space was created through an iterative process of image reconstruction and measurement of subjective similarity, such that the angular separation on the shape wheel corresponds to human similarity judgment. This standardized circular shape space thus provides a simple and parametric space to explore the selection profile.

**Fig. 1:**
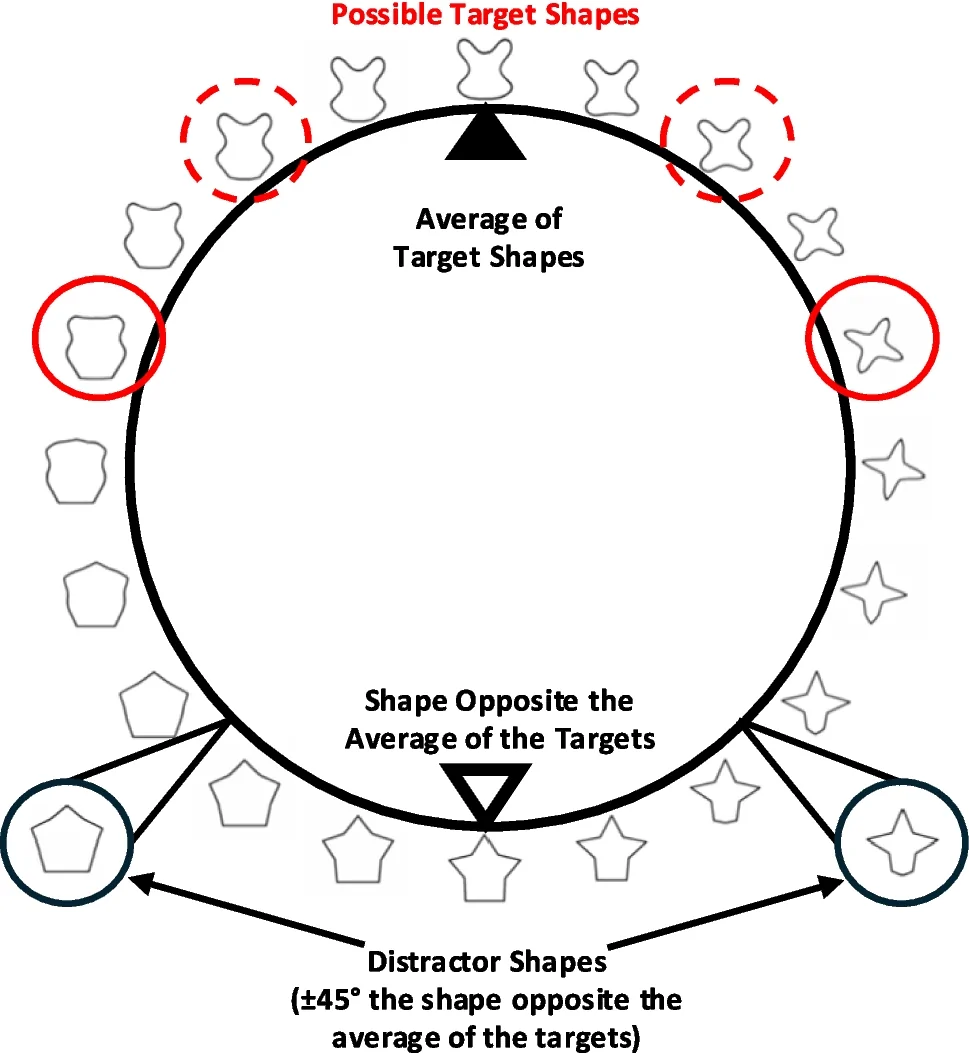
Depiction of the shape space and stimulus selection. The entire shape wheel is shown with a subset of shapes 18° apart. The two shapes in the dashed red circles are 36° apart and those in solid red circles are 72° apart. These pairs could serve as cued target shapes on different trials. Both example sets of target shapes have the same average shape, indicated by the solid triangle. The distractor shapes are selected by first determining the shape opposite this average shape, indicated by the hollow triangle, and then choosing the shapes equal to + 45° and − 45° away from this shape. The exact distractors that would be shown for these targets are in the black circles, since they cannot be fit on the wheel with an 18° spacing. The distractor shape overlaps the target that is most distant from it on the shape wheel. Thus, the distractor shape in the lower left quadrant would overlap with those in the upper right quadrant and vice versa for the ones in the lower right quadrant. (Color figure online)

A limitation with this synthetic approach is that the stimulus set is restricted to a class of 2D closed shapes instead of natural objects with multiple feature dimensions like color, texture, and shading. While this limited stimulus set does not permit us to measure full-fledged object-based selection, we believe that measuring shape-based selection would still be informative about the former. First, shape is far more complex than simple features and is the most important property that defines an object (Baker et al., 2020; Biederman, 1987; Elder & Velisavljević, 2009; Lloyd-Jones & Luckhurst, 2002). Indeed, in this particular shape space, shape is equivalent to object identity. Second, we note that most classical studies on object-based attention used simple outline shapes as carriers of object-hood (Duncan, 1984; Egly et al., 1994; Roelfsema et al., 1998; Vecera & Farah, 1994), demonstrating the utility of using simple shapes as a proxy for visual objects. Thus, while object shape can be construed as a high-level visual feature, studying its selection profile goes beyond simple features and could provide a useful initial step toward understanding object-based attention.

The current study thus aims to identify the selection profile for object shape in the calibrated circular shape space of Li et al. (2020). We adapted the paradigm used in a color-based attention study (Störmer & Alvarez, 2014) by presenting two shapes from the shape space with their differences systematically manipulated. In addition, two additional distractor shapes were overlaid on the two target shapes. Participants performed a change identification task on the target shapes. We hypothesized that participants’ performance on the task would predictably change as the difference between two attended shapes varied, revealing the selection profile for objects. High performance is expected when the two shapes are similar enough to receive prioritized processing (enhancement) without conflicting with each other, whereas low performance is expected when the two objects are dissimilar such that they mutually inhibit each other (suppression). In particular, we were interested in finding out whether a monotonic, shape-similarity gain effect, or a nonmonotonic, surround suppression effect, emerges from our experiments, thus informing us about the underlying mechanism of shape-based selection.

## Experiment 1

### Methods

#### Participants

We performed a power analysis to inform our choice of sample size. Based on our hypotheses, there would be three critical comparisons. The first would be to test for a decrease in performance from when the targets matched to when they were the most different to support a similarity gain structure. The second and third would rely on the presence of a local minimum in the selection profile that could be tested against the target match condition and a subsequent rebound to find evidence for surround suppression. Previous literature suggests that the smallest effect is the difference between the suppression point and rebound (Fang & Liu, 2019; Fang et al., 2019). Hence, we used the effect size from feature-based studies finding surround suppression to guide the choice of sample size. An a priori power analysis for a paired-sample *t* test was conducted using G*Power (Version 3.1.; Faul et al., 2007) based on data from Störmer and Alvarez (2014), which reported an effect size of η^2^ = 0.28 (Cohen’s *d* = .61) for the suppression to rebound comparison, a medium effect using criteria from Cohen (1988). With a significance criterion of α = .05 and power = .80, the minimum sample size needed with this effect size is *N* = 24. We thus recruited 26 participants (16 women, nine men, mean age = 28 years) from the community of students at Michigan State University as well as members of the general East Lansing community. All participants had normal or corrected-to-normal vision and were compensated at a rate of $12 per hour. Informed consent was obtained from every participant. All experimental protocols were approved by the Institutional Review Board at Michigan State University.

#### Apparatus

The experiment was implemented in MATLAB (MathWorks, Natick, MA) with the MGL toolbox (Gardner et al., 2018). The stimuli were presented on a 34-in. LCD display (2,560 × 1,080 pixels, 60-Hz refresh rate) at a viewing distance of 50 cm. The gamma for the monitor was set at 2.2 to approximately linearize the display luminance.

#### Stimulus

The stimuli were composed of 360 closed line drawings of 2D shapes from Li et al. (2020). We made small adjustments to the aspect ratios of the original images so that the overlap between each line drawing was minimized. This was done to make the targets and distractors easier to differentiate when overlapped. The background was set to a mid-gray value of ~ 75 cd/m^2^ and the contours of the shapes were presented at a grayish-white value of ~ 123 cd/m^2^ so that their luminance could be changed to become either brighter or dimmer. On each trial, one target shape was chosen randomly from the wheel while the other was chosen from a ± 0–90° range in increments of 18° (0°, 18°, 36°, 54°, 72°, 90°) from the first shape. We refer to the circular distance between the two targets as “target offset.” There were a total of six offsets based on the angular difference between the two target shapes. Note that although there was directionality (clockwise vs. counterclockwise) on the shape wheel from the viewpoint of a single shape, when paired, all possible target shape pairs for clockwise directions (positive offset) were identical to the possible target shape pairs in the corresponding counterclockwise directions (negative offset). Therefore, it was not meaningful to distinguish the directionality of offset. The distractor shapes were chosen with two steps. First, the midpoint of the two target shapes was calculated and the opposite (180°) location on the wheel was identified. Then, two shapes that were ± 45° away from this location were chosen as distractors and assigned in a way that each target was overlapped with the more distant distractor (see Fig. 1 for a visual example of distractor selection).

#### Procedure

**Fig. 2:**
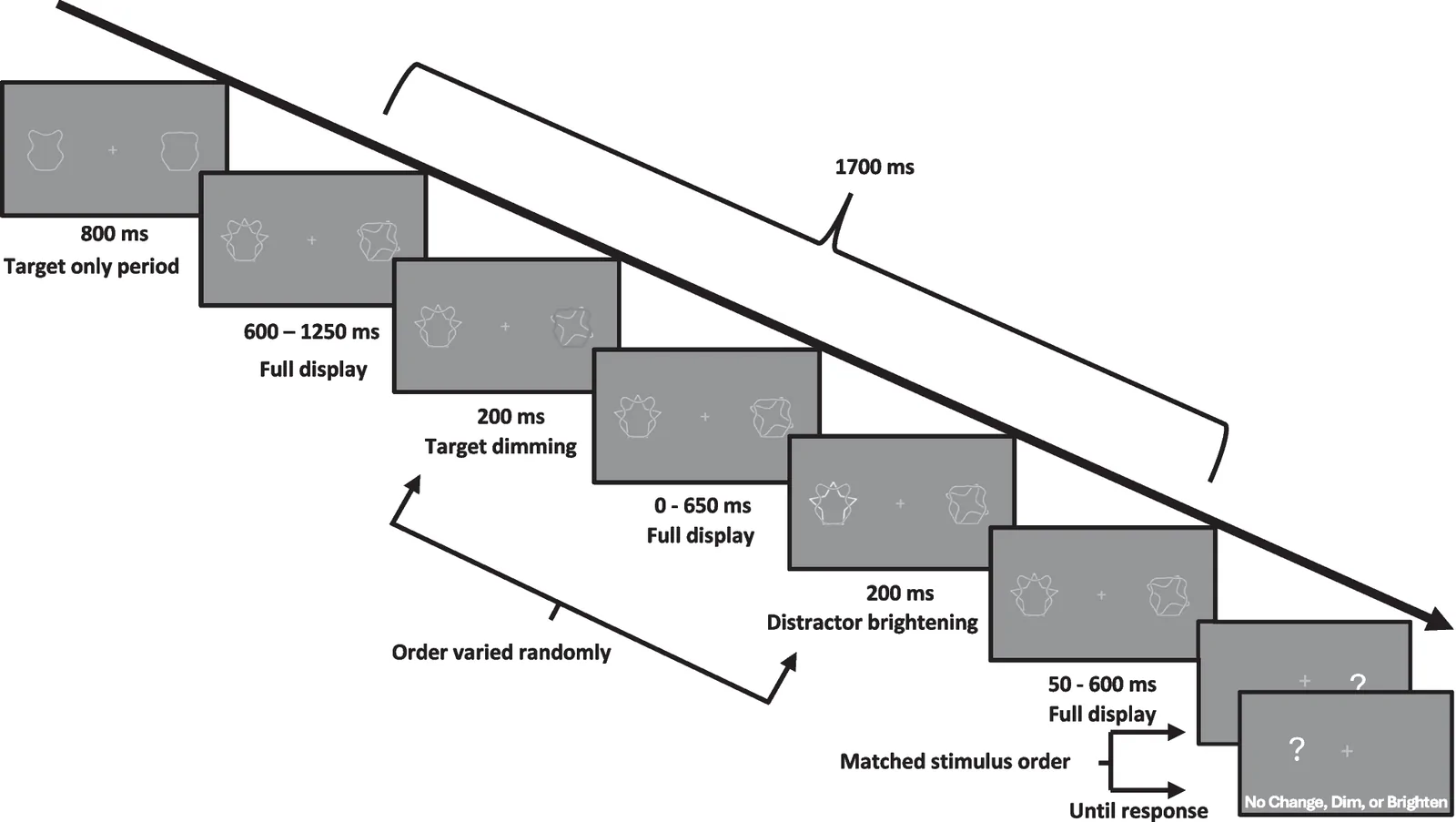
The time course of one trial of the task in both experiments. The two target shapes are displayed for 800 ms before being overlapped by the two distractor shapes. This display persists for 600–1,250 ms until a luminance change occurs. The first luminance change occurs for one shape in one target–distractor pair for 200 ms before being followed by a return to baseline luminance for 0–650 ms. A second 200-ms luminance change then occurs in one shape in the other target–distractor pair before another period of 50–600 ms with the original shapes. It is possible that neither the target nor distractor undergo a change in either pair on a trial, in which case the 200 ms epochs would be identical to the preceding and following epochs. All four shapes appear on the screen together for a total of 1,700 ms. Two prompts are shown sequentially to collects responses at the end of each trial. Upon response, the prompt moves to the other target shape’s location until a second response is made. On this example trial, the right target dimmed first, then the left side distractor brightened. Therefore, the response prompt appeared on the ride side first (correct response: dimmed [press 2]), followed by the response prompt on the left side (correct response: no change [press 1]).

The timing of a single trial is shown in Fig. 2. On each trial, participants were presented with two target shapes located 3.25° to the left and right of a central fixation cross (size: 0.44°) which was present throughout the trial (Fig. 2). Participants were instructed to pay attention to the target shapes and only respond to changes in these shapes. These shapes were presented for 800 ms before two distractor shapes appeared at the same locations, which the participants were told to ignore. All four shapes were shown on the screen for 600–1,250 ms before a brief 200-ms luminance change occurred for one of the shapes in one of the target–distractor pairs, followed by another 200-ms luminance change for a shape in the other pair. The order in which the luminance changes occurred (left or right side first) was randomized throughout all trials. The time interval between the two luminance changes randomly varied between 0 and 650 ms, and the luminance changes were followed by another interval of 50–650 ms before the shapes disappeared. All four shapes were present on the screen for a total of 1,700 ms on each trial. The shape pairs were then replaced by a response prompt that indicated which target shape to respond to. The participants then reported whether the target shape on that side did not change, became dimmer, or became brighter. After the first response, the prompt then moved to the other side and participants responded again. The order in which the response prompt appeared always matched the order in which luminance changes occurred. This was done to reduce memory load that could lead to confusion in the response stage of the task. For both left and right shapes, independent of each other, the distractor dimmed or brightened in ~30% of trials, the target dimmed in ~30% of trials, the target brightened in ~ 30% of trials, and no change occurred in ~10% of trials.

The participants performed this task in three phases of a single session. First, participants were given the opportunity to practice the task to acclimatize themselves to the procedure. Once they indicated to the experimenter that they understood the task, they began a thresholding task. The thresholding task utilized two separate 2-down 1-up staircases (Levitt, 1971) on dimming and brightening trials, respectively, which were used to adjust the magnitude of the luminance changes of the shapes to achieve ~71% accuracy. Participants performed at least three blocks, 60 trials each, of this task, until both diming and brightening staircases stabilized. During the main task phase, the luminance change values were fixed at the individual participant’s threshold. They then completed four blocks of 120 trials resulting in a total of 480 trials per participant. Given that participants made two independent responses on each trial, we obtained 960 responses in total, resulting in 160 responses for each of the six offset conditions.

#### Data analysis

**Fig. 3:**
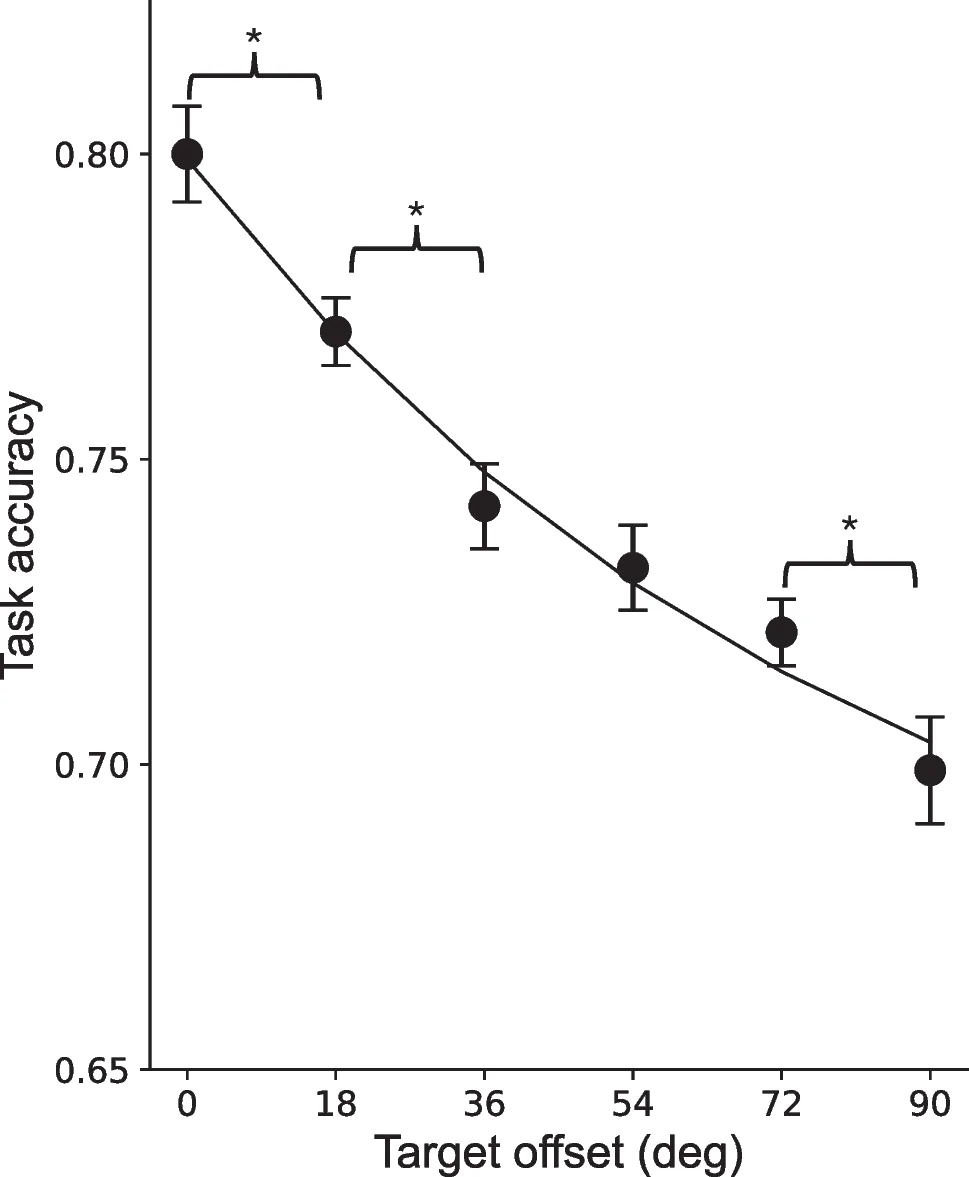
Results for Experiment 1. The data points represent group average accuracy for each target offset. The error bars represent estimated within-subject error using the method of Cousineau (2005). The line shows the best fitting exponential model. Asterisks indicate a statistically significant difference.

**Fig. 4:**
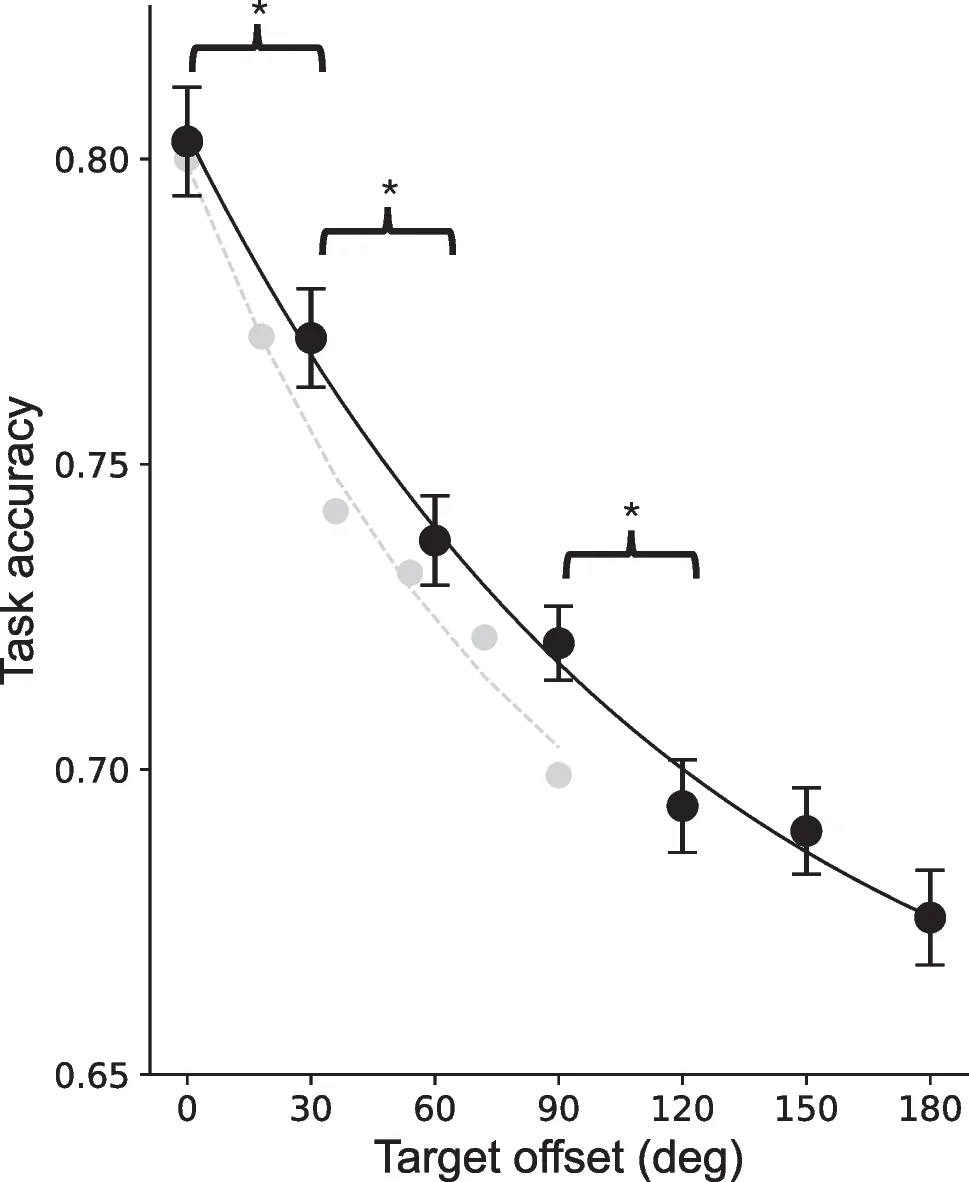
Results for Experiment 2. The black data points represent group average accuracy. The error bars represent estimated within-subject error using the method of Cousineau (2005). The solid curve shows an exponential model fit to the data. The light-gray data points and the dashed line replot the data from Experiment 1 for comparison. Asterisks indicate a statistically significant difference.

To evaluate how shape offset impacts task performance, participant task accuracy for each of the six target offset conditions was compared. All statistics were calculated using custom code in MATLAB and data visualization was created using Python. The error bars displayed in Figs. 3 and 4 are created using the method in Cousineau (2005) for calculating within-subject error. An exponential fit was plotted in each figure based on the best fitting model of group data (see Supplemental Materials, Model Fitting).

A one-way repeated-measures analysis of variance (ANOVA) was used to test whether the general trend observed was significant. For a monotonic or nonmonotonic selection profile, a main effect of target offset would be expected, with the 0° offset condition having the highest accuracy. Planned comparisons were then performed on adjacent offset conditions to test for significant (α = .05) condition-by-condition fluctuations. This analysis would be most informative if a nonmonotonic pattern was observed. In addition to this analysis on task accuracy data, we also performed analyses using the signal detection theory, as well as analyses to examine the influence of secondary factors such as luminance change direction, target response order, and relative location on the shape wheel (reported in Supplemental Materials).

### Results and discussion

We calculated the proportion of correct responses for trials in each target offset condition. We observed a general decrease in task accuracy as the two target shapes become more different (Fig. 3). A one-way repeated-measures ANOVA was conducted to assess the effect of shape offset on task performance, which revealed a significant main effect of target offset, *F*(5,125) = 21.22, *p* < .001. Follow up comparisons were conducted between each adjacent target offset condition to test the significance of the decreasing trend. Significant differences were found between the 0° and 18° conditions, *t*(25) = 3.73, *p* < .001, *d* = 0.73, CI [0.013, 0.045], 18° and 36° conditions, *t*(25) = 3.125, *p* = .004, *d* = 0.61, CI [0.009, 0.047], and the 72° and 90° conditions, *t*(25) = 2.30, *p* = .030, *d* = 0.451, CI [0.002, 0.043]. While the other comparisons did not reach statistical significance, they all exhibited a decreasing trend. We did not observe numerical rebound anywhere that would indicate surround suppression. This general trend in performance was also found when the analysis was conducted using sensitivity (*d′*) in place of raw task accuracy (see Supplemental Materials, Fig. S1) as well as conditioned on secondary factors (see Supplemental Materials, Fig. S2–S5).

In summary, in Experiment 1, we examined the effect of manipulating the distance between two target shapes on performance on a luminance change identification task. We found the highest accuracy when the two targets matched (i.e., 0° offset), with decreasing accuracy as the difference between the two target shapes grew. This suggests that when attending to two shapes within this shape space, there is interference that makes it harder to select two distinct shapes. In sum, these findings support both the idea that, to observers, the shapes differ from each other parametrically and that observers’ performance, in turn, fluctuates in a predictable manner as the shapes become more different, rather than randomly, as would be predicted if the shape differences were orthogonal to attentional processes.

While these results show a monotonic selection profile more in line with the feature similarity gain model, there is a limitation in this interpretation because we only tested shapes in a limited range (0–90° offset on the wheel). This leaves open the possibility that a different pattern of results could be observed for more distinct shapes beyond this range, if, for example, a rebound in performance (consistent with a surround suppression effect) occurs between 90° and 180° offset. Thus, we conducted a second experiment, by widening the range of possible target differences to 180° (i.e., maximal difference on the wheel), to test whether this pattern would be maintained for the full range of difference within the shape wheel.

## Experiment 2

### Methods

The same a priori power analysis and participant requirements from Experiment 1 were used to guide Experiment 2. Participants (*N* = 26, 13 women, 12 men, mean age = 28 years) came from the community of students at Michigan State University as well as members of the general East Lansing community and were compensated at a rate of $12 per hour. Informed consent was obtained from every participant.

The same materials, stimuli, procedure, and analysis were used in Experiment 2 as in Experiment 1, except for one key difference. The target shapes were chosen to be ± 0–180° different on the shape wheel in increments of 30° (0°, 30°, 60°, 90°, 120°, 150°, 180°). This resulted in seven target offset groups in Experiment 2. Here, participants performed three blocks of 63 trials in the thresholding task and four blocks of 140 trials in the main task. This resulted a total of 560 trials per participant and with two responses per trial, resulted in 160 responses per target offset condition for a total of 1,120 responses per participant.

### Results and discussion

The proportion of correct responses as a function of shape offset shows a similar monotonic pattern as in Experiment 1 (Fig. 4). A one-way repeated-measures ANOVA confirmed that there was a main effect of target offset, *F*(6,150) = 30.82, *p* < .001. Follow-up comparisons were conducted between each adjacent target offset condition to test the significance of the decreasing trend. A significant difference were found between the 0° and 30° conditions, *t*(25) = 3.54, *p* = .002, *d* = 0.69, CI [0.013, 0.051], the 30° and 60° conditions, *t*(25) = 2.82, *p* = .009, *d* = 0.55, CI [0.009, 0.057], and the 90° and 120° conditions, *t*(25) = 2.86, *p* = .008, *d* = 0.56, CI [0.007, 0.046]. While the other comparisons did not reach statistical significance, all exhibited a decreasing trend. We again did not observe any numerical rebound anywhere that would indicate surround suppression. This general trend in performance was also found when the analysis was conducted using sensitivity (*d′*) in place of raw task accuracy (see Supplemental Materials, Fig. S1) as well as conditioned on secondary factors (see Supplemental Materials, Fig. S2–S5).

In Experiment 2, we examined the effect of shape offset between two targets in the same luminance change identification task across the whole range of possible differences in the circular shape space. We again observed a monotonic performance pattern such that accuracy decreased as the difference between the two target shapes grew without any apparent rebound in performance. Because the widened range in Experiment 2 included that of Experiment 1, this also offers an opportunity to replicate the results from Experiment 1. We replotted Experiment 1 data in Fig. 4 (light-gray plot) that covered the 0 to 90° range. Within this range of shape offset, the results from both experiments strongly resembled each other, with similar overall accuracy level and a similar rate of decline. Beyond this range, it appeared that the drop-off of the shape-similarity gain effect continued as a function of target shape without a change in the monotonic pattern. This shows that the results from Experiment 2 replicate and extend the results from Experiment 1, suggesting a similarity gain profile across the entire shape space.

One possible limitation of our design is that shape offset (i.e., target–target similarity) is not entirely independent of the target–distractor similarity, raising the possibility that the latter was driving the observed effects. In our design, as the two target shapes became more dissimilar, their relationship with their overlapped distractor also changed. However, it is important to note target–distractor similarity was not a monotonic function of shape offset. When shape offset increased from 0° to 90°, target and distractor became more distinct, with their angular separation increasing from 135° to 180°. In contrast, when the shape offset further increased from 90° to 180°, target and distractor became more similar, as their separation decreased from 180° back to 135°. Thus, target–target and target–distractor similarity were not inversely related across the full range of offsets. Critically, the data from the 0°–90° and from 90°–180° ranges exhibited highly similar monotonic trends, despite the reversal in the target-distractor similarity function. This pattern makes it unlikely that target–distractor similarity is primarily responsible for the observed similarity-gain effect. Nevertheless, future research should more directly disentangle the respective contributions of target–target and target–distractor similarity to the selection profile for shapes.

## General discussion

In both experiments, we found a monotonic decline in performance as the two target shapes became more different. This pattern of results obtained in the artificially constructed space parallels that from simple feature spaces that support a feature-similarity gain profile (Bondarenko et al., 2012; Martinez-Trujillo & Treue, 2004; Wang et al., 2015). The shape space used here was created from subjective similarity judgments (Li et al., 2020). However, in our task, participants never made any explicit similarity judgment; they were simply instructed to identify the changes that occurred on two target shapes. Thus, the finding that performance fluctuates predictably with the difference in similarity between the two attended shapes suggests that attention operates with a gradient-like structure in an intrinsic similarity space that goes beyond simple features. This is interesting since this similar pattern of results is present for simple feature (Liu & Hou, 2011; Martinez-Trujillo & Treue, 2004) and location-based attention (Downing, 1988; Handy et al., 1996; Posner et al., 1980), suggesting a common mechanism across all three domains. We consider this selection profile to be shape-similarity gain, analogous to feature-similarity gain.

This shape-similarity effect suggests that, even though these shapes were likely quite novel to the participants, they were nevertheless tuned in to the underlying similarity structure. This result can be interpreted in the broad framework of prototype theory of categorization (Hampton, 1995; Rosch & Mervis, 1975), where objects are represented by their similarity to a central prototype. Although theories of categorization are concerned primarily with real-world objects with multidimensional attributes, analogous theories for novel visual objects are also possible. For example, Edelman (1998) proposed that we represent objects, not by giving each object its own unique representation but, by comparing the internal representation of a viewed stimulus with neural representations of a library of reference objects. This theoretical approach postulates that the more distant a perceived shape is from an external reference in a parameter space, the more different the internal mental representation of that perceived shape is from the internal reference shape, creating a monotonic decline in overlap that allows us to classify shapes based on their distinctness from these references. It is possible to reconstruct the similarity structure of a complex parameter space in a faithful way based on human subjects’ psychophysical data (Cutzu & Edelman, 1998), supporting the physiological plausibility of such a representational scheme for object spaces. Consistent with this, for the stimulus space used in our experiment, just like with any novel stimulus we might come across, we expect that some component of its internal representation can be compared to our prior experiences. Our results support a similar mechanism of attention that is sensitive to the parameter-space distance of the two external shapes and produces a monotonic decline in performance as this difference grows, suggesting a more taxing attentional process when two very different sets of internal units need to be modulated by attention.

Our results naturally prompt further consideration of the mechanisms driving the observed pattern. We note that our task design encourages attending to the two target shapes simultaneously, given that the temporal order of the luminance change events was randomly determined on each trial. However, because our task is somewhat complex (two change events in two hemifields and two responses), variations of task strategies over time could potentially explain our results. One possibility is that it is easier to attend to two shapes simultaneously when they are similar but as they become more different, participants adopt a more serial strategy to attend one at a time. In particular, given the general leftward bias in visuospatial processing (Jewell & McCourt, 2000; Rinaldi et al., 2014), participants could prioritize the left target more when the two targets become more different. There is some limited evidence for such a mechanism when we analyzed data by target hemifield (see Supplemental Materials, Fig. S5). However, we consider the evidence anecdotal due to a relatively weak effect and lack of quantitative modeling (see Supplemental Materials for more details). Nevertheless, these observations rule out extreme models of a serial strategy such as attending only to the left target and ignoring the right target, which would predict chance-level performance for the right target at large target offsets, an effect clearly not observed. While the possibility of a hemifield-based mechanism underlying the observed selection profile is certainly intriguing, we remain cautious in such an interpretation. Future research with richer experimental manipulations and quantitative modeling is needed to determine the temporal dynamics of attentional allocation in this task. Regardless of the underlying attentional dynamics, the shape-similarity gain effect is robust in all the additional analyses conditioned on secondary factors (see Supplementary Materials), reinforcing this effect as a strong component of the selection mechanism.

It is also interesting to consider the implications of our results for the underlying neural mechanism. While it may seem that we need incredibly vast and complex neural structures to represent all possible complex objects, there may be a simpler set of dimensions encoded by our brains that help represent this nearly infinite space. One example of such a functional organizational principle in the primate visual cortex is topography, that is, adjacent neurons in cortex process similar information. This is true for spatial locations (i.e., retinotopy) as well as feature dimensions such as color, orientation, and numerosity (Harvey et al., 2013; Li et al., 2014; Tanigawa et al., 2010; Xiao et al., 2003). This topographical arrangement would be a natural substrate to generate a similarity-gain effect as top-down attentional modulation is likely subject to anatomical and structural constraints. Under this framework, our observed shape-similarity gain effect would imply a similar organizational principle exists for objects. Although classical work on object recognition has focused on category-selective responses in high-level visual areas such as selectivity for faces, places, and words (Cohen et al., 2000, 2002; Epstein & Kanwisher, 1998; Kanwisher et al., 1997), more recent work with a data-driven approach has shown that neural responses in these areas can be projected to a reduced dimension space with meaningful axes (Bao et al., 2020; Chang & Tsao, 2017; Yao et al., 2023). For example, recording from monkey inferotemporal cortex, Bao et al. (2020) found that the first two principal dimensions to which neuronal responses are sensitive can be construed as animate/inanimate and spikey/stubby. Importantly, this two-dimensional object space seems to be organized similarly to the way simple features are organized in early visual areas with spatially repeating neuronal clusters representing visually similar information (Li et al., 2014; Tanigawa et al., 2010; Xiao et al., 2003)—that is, a topographic organization. Initial human neuroimaging studies have also reported analogous results along this spikey–stubby axis (Coggan & Tong, 2023; Yargholi & de Beeck, 2023). Relatedly, we note that the shapes used in our study can be considered as varying along this spectrum from “spikey” to “stubby” (see Fig. 1). Thus, it is plausible that differences between representations of a shape stimulus and representations of prototypical “spikey” shapes and “stubby” shapes forms the substrate on which attentional selection occurs in our task. While still in early stages, we nevertheless found the convergence of diverse lines of research quite intriguing and suggestive for future investigations of the neuronal basis of feature and object-based attention.

We did not find evidence for a surround suppression profile in our experiments. Given that our experimental paradigm followed that of a previous study (Störmer & Alvarez, 2014), which reported a surround suppression effect for color, this could suggest that surround suppression does not occur for simple objects. We believe that this discrepancy could be caused by the increase in complexity from simple features to shapes. According to one explanation of surround suppression, the Selective Tuning model (Cutzu & Tsotsos, 2003; Tsotsos, 2011), attention operates via a top-down, hierarchical selection process where cortical feedback narrows down potential targets and suppresses the neural representations of nearby, irrelevant information. As feedback continues through layers of decreasing complexity, more neural representations are attenuated and this continues until there is only one representation, ideally the target, remaining unattenuated (Tsotsos, 2011). This process can produce a suppressive surround for both spatial and feature-based selection. Because shapes are processed at a higher level of the visual hierarchy than simple features, it could be that there is not enough depth in the cortical hierarchy to allow such progressive feedback to generate a suppressive surround. Alternatively, there are other differences between our experiments and those that reported surround suppression effects for simple features. One salient difference is the lack of familiarity with the stimulus set in our experiments. Unlike the feature spaces of simple features, the shape space used here is highly novel to participants such that participants potentially could not effectively evaluate similarity. For example, we experience colors nearly constantly and color categories appear to be acquired at a very young age (Bornstein et al., 1976; Skelton et al., 2017). It is possible that more familiarity with the shapes would attenuate reasonably similar distractors and suppress them. This seems plausible as we know that familiarity and expertise with a class of visual stimulus can change their neural representations in high-level visual cortical areas (Harel, 2016; Hoffman & Logothetis, 2009; Kakaei & Braun, 2024). Relatedly, familiarity may further lead to categorical representations, and we note that a previous study reported that suppressive surrounds for colors coincided with categorical boundary (Fang et al., 2019). Thus, familiarity and possibly category formation may ultimately lead to a surround suppression effect in the shape space. Further research is needed to examine how the selection profile for visual objects is influenced by these factors.

Finally, although we are not testing full-fledged object-based attention, we believe that our study takes an effective first step towards object-based attention, given the prominence of shape in objecthood. To place this in a real-world context, when searching for an object, our expectations of the target shape could differ from a suitable target object in our view and thus inhibits its processing, as in the case of searching for milk in the refrigerator and missing the carton of milk because our search template is for a jug of milk. This suggests that a potential way to ameliorate the frustration of difficult search tasks in our daily life is to ensure we have accurate and descriptive information about a target prior to search. At a minimum, our study provides a useful link between existing research on feature-based attention and future research in object-based attention with more realistic stimuli and higher ecological validity.

In conclusion, we found that attentional selection of shapes follows a shape-similarity gain profile in the artificially constructed shape space. We believe that this pattern represents the selection profile for more complex stimuli than previously tested and approaches a profile for simple object-based attention. Our results suggest that attention operates on internal representations that encodes a systematic similarity structure. These results set the stage for future research on the influence of stimulus and task factors on the selection profile, as well as the underlying neural mechanisms.

## Supplementary Information

Below is the link to the electronic supplementary material.

Supplementary file1 (DOCX 1.52 MB)

## Declarations

### Open practices statement

The trial level data for all participants in both experiments as well as respective analysis code is available online (https://osf.io/hz7uc/). Stimulus code can be made available upon request. These experiments were not preregistered prior to being conducted.
