# Supplementary material for: Shape-similarity gain: A selection profile for simple objects
Source: Atten Percept Psychophys. 2026 Jun 9;88(5):141. doi: 10.3758/s13414-026-03283-y (PMC13249725; doi:10.3758/s13414-026-03283-y)
Supplement: Supplementary file 1 — Supplementary file1 (DOCX 1.52 MB) [file 13414_2026_3283_MOESM1_ESM.docx]

**Supplemental Information**

**Shape-similarity gain: a selection profile for simple objects**

Brendan Valentine^1^, Xiaoli Zhang^1^, Taosheng Liu^1^

*^1^Department of Psychology, Michigan State University, East Lansing, MI*

**Supplemental Data Analysis**

***Model fitting***

We tried to characterize the relationship between shape offset and performance using a model fitting approach. Given that our results showed a monotonic decline of performance as a function of shape offset, we chose three monotonic functions, a linear, a Gaussian, and an exponential function, to fit the accuracy data. The linear model takes the following functional form:

$$P_{c}=ax+b$$

where *a* and *b* are free parameters of the linear function, *x* is the target offset, and *P_c_* is the task accuracy. A linear profile would be expected if task performance was directly related to the perceptual similarity between shapes, which defines the shape space (Li et al., 2020). The Gaussian model takes the following functional form:

$$Pc=\frac{a}{w}e^{- \frac{x^{2}}{{2w}^{2}}}+ b$$

where *a,* *w*, and *b* are free parameters. Here, *a* and *w* control the shape of the function, and *b* controls the asymptotic value. The Gaussian model has been used to fit data from previous study on the selection profile of simple features (Fang et al., 2019). The exponential model takes the following functional form:

$$P_{c}=a b^{x}+c$$

where *a*, *b*, and *c* are the free parameters of the exponential function and *x* is the target offset. Here, *a* and *b* control the shape of the exponential function and *c* controls the asymptotic value. An exponential model was chosen because it has been proposed to reflect a universal law that relates behavioral generalization to distance in a psychological space (Shepard, 1987; Sims, 2018). To compare these models, a Bayesian information criterion (Raferty, 1995, 1999; Wagenmakers, 2007) is calculated for each model from the residual sum of squares and then the two best fitting models are used to produce a Bayes factor (Raferty, 1995, 1999), where a factor greater than one supports the first model and vice versa.

In Experiment 1, the group-averaged data was best fit by an exponential model (R­^2^ = .985), with a Bayes factor of 6.18 over the next-best fitting linear model (R_­_^2^ = .962). However, at the individual level, the linear model is preferred for most participants (Table S1), which suggests a model identifiability problem. For Experiment 2, we observed a similar pattern, where an exponential model had the best fit for the group average data (R_­_^2^ = .994) with a Bayes factor of 220.69 over the next best-fitting Gaussian model (R_­_^2^ = .974), but the linear model was the most favored in individual participant fits (Table S1). These discrepancies of model fit prevent us from determining the precise functional forms of the selection profile. However, given that the primary objective of the current study is to establish the general shape of the profile (i.e., monotonic vs. non-monotonic), we plotted the group data with an exponential fit for visualization purpose only. Future research with more data and more sophisticated statistical procedures could shed light on the precise functional form of the selection profile.

***Signal detection analysis***

In the main analysis, we used correct identification of the luminance changes to measure performance. As a complementary analysis, we also performed signal detection analysis, focusing on the detection aspect of our task, i.e., participants first need to detect a change on the target, regardless of its sign, as opposed to a change on the distractor. Thus, we can define hit as selecting that any target luminance change occurred regardless of its sign (dimming vs. brightening), and false alarm as selecting a luminance change occurred when either a distractor changed, or no change occurred.

In Experiment 1, we calculated sensitivity (*d’*) and criterion (*C*) for target offset conditions 0° – 90° in increments of 18°. The sensitivity decreased in a monotonic pattern as target offset increased and the criterion value was slightly negative but stable across target offsets, suggesting a slightly liberal response when reporting the presence of a change (Figure S1, left column). The hit rate and false alarm rate (conditioned by whether the distractor changed or no change occurred) are shown in Table S2, which demonstrates that the observed decrease in sensitivity with larger offset is due to a combination of a decrease in hits and an increase in both types of false alarms. A one-way repeated-measures ANOVA on the *d’* revealed a significant effect of target offset, *F*(5,125) = 24.59, *p* <.001. We did not observe numerical rebound that would indicate surround suppression. This pattern of results mirrors that from the main analysis based on accuracy. A one-way repeated-measures ANOVA on the criterion found no significant effect of target offset, *F*(5,125) = 0.57, *p* =.721.

In Experiment 2, sensitivity also decreased in a monotonic pattern as target offset increased and the criterion value was slightly negative and largely stable across target offsets, (Figure S1, right column). A similar break-down of the proportion data as in Experiment 1 is shown in Table S3, which again shows that the observed decrease in sensitivity is due to a combination of a decrease in hits and an increase in both types of false alarms. A one-way repeated-measures ANOVA on *d’* revealed a significant effect of target offset, *F*(6,150) = 31.61, *p* <.001. We did not observe numerical rebound that would indicate surround suppression. This pattern of results is also consistent with that from the main analysis based on accuracy. Somewhat surprisingly, a one-way repeated-measures ANOVA on the criterion measure revealed a significant effect of target offset, *F*(5,125) = 2.37, *p* =.033. This appears to be related to unsystematic changes in the middle of the offset range (e.g., 90 – 150°). Because there was no general trend in the criterion data, follow up comparisons were not performed.

The trend of a monotonic decrease in sensitivity as target offset increased matches the trend of the accuracy data reported in the main paper. This reinforces the idea that it is easier to notice and report a luminance change in a target when the two target shapes are more similar. Because this sensitivity measurement does not count a mistake in tracking the sign of luminance change as an error, this analysis rules out contribution from potential misunderstanding of the task, thus further strengthening the main findings of the study. The stable and slightly liberal response criterion is unsurprising since target change occurred on more than half of the trials (60%). Overall, this analysis provides further insight into the effect observed in the main analysis, using an alternative and complementary measure of task performance.

***Assessing task difficulty for direction of luminance change***

Given the task required participants to identify either a brightening or dimming event, we separated responses by luminance change direction and examined the effect of target offset on task performance. In Experiment 1, the same general monotonic pattern as in the aggregate data (Figure S2, left panel) was found for both target types with a main effect of target offset, *F*(5,125) = 18.40, *p* <.001, with no other significant effects from a 6 × 2 repeated-measures ANOVA. While there was numerical increase in performance between the 54° and 72° for the dimming target and the 36° and 54° for the brightening target, these difference were not statistically significant (*p*s > 0.7, paired t-test) and therefore did not indicate a surround suppression profile. In Experiment 2, the same general monotonic pattern as in the aggregate data was found for both target types while the task was slightly easier for dimming targets (Figure S2, right panel). A 7 × 2 repeated-measures ANOVA revealed a main effect of target offset, *F*(6,150) = 30.46, *p* <.001, and a significant effect of luminance change direction, F(1,25) = 4.29, p =.049. Importantly, there was no interaction effect between target offset and luminance change direction in either experiment. These findings suggest that the effect of target offset on task performance was similar for both luminance change directions.

***Analysis of the data by response order***

We assessed whether response order modulates the effect of target offset, as it is possible that noise in memory and response generation could differentially modulate the observed gain effect. After parsing our data by first and second responses, we found the same general pattern of monotonic decline as in the aggregate data for both response order conditions in both experiments (Figure S3). In Experiment 1, a 6 × 2 repeated-measures ANOVA revealed main effects of target offset, *F*(5,125) = 21.22, *p* <.001, and response order, *F*(1,25) = 19.20, *p* <.001, as well as a significant interaction effect, F(5,125) = 2.48, p =.035. While there was numerical increase in performance between the 54° and 72° offset for first responses and between the 36° and 54° offset for second responses, these were not statistically significant (*p*s > 0.6, paired t-test) and therefore did not indicate a surround suppression profile. In Experiment 2, a 7 × 2 repeated-measures ANOVA revealed significant main effects of target offset, *F*(6,150) = 30.82, *p* <.001 and response order, *F*(1,25) = 9.02, *p* =.006, with a marginally significant interaction effect, F(6,150) = 1.88, p =.087. No numeric trend of rebound between adjacent offsets was apparent.

While the first luminance change target is reported at a higher accuracy overall, the difference between the first and second target appears to grow larger as the two shapes become more different. However, the statistical reliability of this interaction effect is rather weak across the two experiments. It is unclear what caused this pattern of results. While this effect could reflect attentional processes (e.g., shifting attention from the first target to the second target), it could also be related to memory processes. For example, better performance for the first target could reflect a primacy effect, and the marginal interaction effect could reflect stronger competition in memory as items become dissimilar. The specific causes of these order effects are beyond the scope of the current study. Importantly, for both first and second responses, monotonic selection profiles were observed, consistent with the result based on all data.

***Consistency of the gain effect around the shape wheel***

In our experiments, we randomly selected target shapes from around the entire shape wheel to comprehensively sample the entire shape space. It may be possible that the pattern of results differs for different subsections of the shape space. To test this possibility, we divided the space into quartiles each 90° wide on the shape wheel (Figure 1) and examined task performance by target offset for each quartile. Although there are many possible ways of subdivision, the quartile division appears to capture qualitative changes in shapes across the space, while maintaining a reasonable number of trials per condition. The two objects on each trial were coded by the quartile to which they belong and also the relative offset between the two shapes. For both experiments, parsing the data in this way showed a general monotonical profile as observed in the aggregate data (Figure S4). For Experiment 1, a 6 × 4 repeated-measures ANOVA revealed a significant main effect of target offset, *F*(5,125) = 20.74, *p* <.001, and quartile, *F*(3,75) = 4.73, *p* =.004, without a significant interaction effect, *F*(15,375) = 1.08, *p* =.377. There was a numerical increase in performance between the 36° and 54° conditions of the first quadrant, which was not statistically significant (*p* = 0.254, paired t-test). Similarly, for Experiment 2, a 7 × 4 repeated-measures ANOVA revealed a significant main effect of target offset, *F*(6,150) = 30.29, *p* <.001, without a significant interaction effect, *F*(18,450) = 1.02, *p* =.436. The numerical increase in performance between the 120° and 150° conditions of the third quadrant was not statistically significant (*p* = 0.079, paired t-test) The lack of a significant interaction effect in these analyses suggests that the similarity gain effect is present to a similar degree across the shape wheel. We note that there is a main effect of quartile from Experiment 1, which raises the possibility that some shapes in the space are easier overall to respond to than others. However, this pattern was not found in Experiment 2, suggesting that it may be not a reliable effect.

***Analysis of the data by target hemifield***

Despite our design encouraging simultaneous attending of both targets, participants may have had a preferred visual hemifield and directed their attention primarily to the target in that hemifield, leaving only leftover attentional resources to respond to the less preferred target. Previous research has found a hemifield preference consistent with reading direction (Rinaldi et al., 2014) as well as a general leftward bias, i.e., the pseudoneglect effect (Jewell & McCourt, 2000). We parsed our data by left and right target responses to determine if our main finding was maintained for both targets and if this analysis was indicative of a task strategy.

The same general pattern of monotonic decline as in the aggregate data was found for both hemifields across both experiments (Figure S5). In Experiment 1, a 6 × 2 repeated-measures ANOVA revealed a significant main effect of target offset, *F*(5,125) = 21.22, *p* <.001 without a significant effect of target hemifield or an interaction effect. In Experiment 2, a 7 × 2 repeated-measures ANOVA revealed a significant main effects of target offset, *F*(6,150) = 30.82, *p* <.001, target hemifield, *F*(1,25) = 14.46, *p* =.001, as well as a significant interaction between target offset and hemifield, *F*(6,150) = 2.56, *p* =.021. The numerical increase in performance between the 120° and 150° left hemifield responses was not statistically significant (*p* = 0.419, paired t-test). Taken together, these results show that there is some evidence for a left hemifield bias (at least in Experiment 2), consistent with previous research (Jewell & McCourt, 2000; Rinaldi et al., 2014). The modest interaction effect in Experiment 2 also shows that this leftward bias is more pronounced as the two attended shapes become more different. Such a pattern is inconsistent with a strategy that participants only attended to the left target and ignored the right target at large offsets, which would predict chance performance for the right target. However, this pattern is consistent with a less extreme scenario where participants were able to attend to both target shapes in parallel when they are similar but, prioritized the left target when the two shapes become more different. While such a scenario is certainly intriguing, our data likely lack the power to identify the precise model underlying the performance. Furthermore, there is some inconsistency in this effect between the two experiments, thus requiring us to be cautious in this interpretation. Importantly for the present purpose, we again observed monotonic selection profiles for both the left- and right-hemifield targets, supporting the main conclusion drawn from the aggregate data.

**References**

Cousineau, D. (2005). Confidence intervals in within-subject designs: A simpler solution to Loftus and Masson’s method. In *Tutorials in Quantitative Methods for Psychology* (Vol. 1, Number 1).

Fang, M. W. H., Becker, M. W., & Liu, T. (2019). Attention to colors induces surround suppression at category boundaries. *Scientific Reports*, *9*(1). https://doi.org/10.1038/s41598-018-37610-7

Jewell, G., & McCourt, M. E. (2000). Pseudoneglect: a review and meta-analysis of performance factors in line bisection tasks. *Neuropsychologia*, 93–110. www.elsevier.com/locate/neuropsychologia

Li, A. Y., Liang, J. C., Lee, A. C. H., & Barense, M. D. (2020). The Validated Circular Shape Space: Quantifying the Visual Similarity of Shape. *Journal of Experimental Psychology: General*, *149*(5), 949–966. https://doi.org/10.1037/xge0000693.supp

Raferty, A. E. (1995). Bayesian Model Selection in Social Research. *Sociological Methodology*, *25*, 111–163.

Raferty, A. E. (1999). Bayes Factors and BIC. *Sociological Methods & Research*, *27*(3), 411–427.

Rinaldi, L., Di Luca, S., Henik, A., & Girelli, L. (2014). Reading direction shifts visuospatial attention: An Interactive Account of attentional biases. *Acta Psychologica*, *151*, 98–105. https://doi.org/10.1016/j.actpsy.2014.05.018

Shepard, R. N. (1987). Toward a Universal Law of Generalization for Psychological Science. *Science*, *237*(4820), 1317–1323.

Sims, C. R. (2018). Efficient coding explains the universal law of generalization in human perception. *Science*. https://www.science.org

Wagenmakers, E. (2007). A practical solution to the pervasive problems of p values. *Psychonomic Bulletin & Review*, *14*(5), 779–804.

**Figure S1 Results from signal detection analysis for Experiment 1 (left panels) and Experiment 2 (right panels)**.Graphs in the top row show the sensitivity parameter (d’). The graphs in the bottom row show the criterion parameter (C). Error bars represent within-subject error as defined by Cousineau (2005).


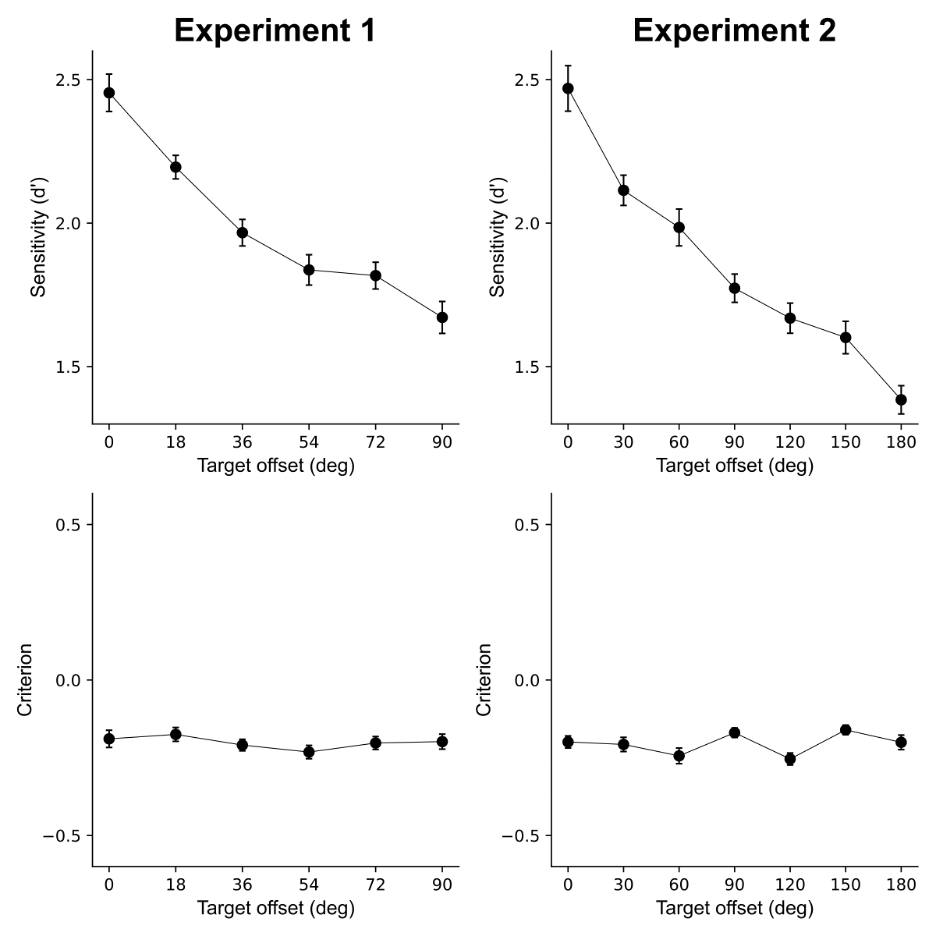


**Figure S2 Results conditioned by the sign of the luminance change in Experiment 1 (left panel) and Experiment 2 (right panel)**.The black lines plot accuracies to dimmed targets while the grey lines plot accuracies to brightened targets. Error bars represent within-subject error as defined by Cousineau (2005).


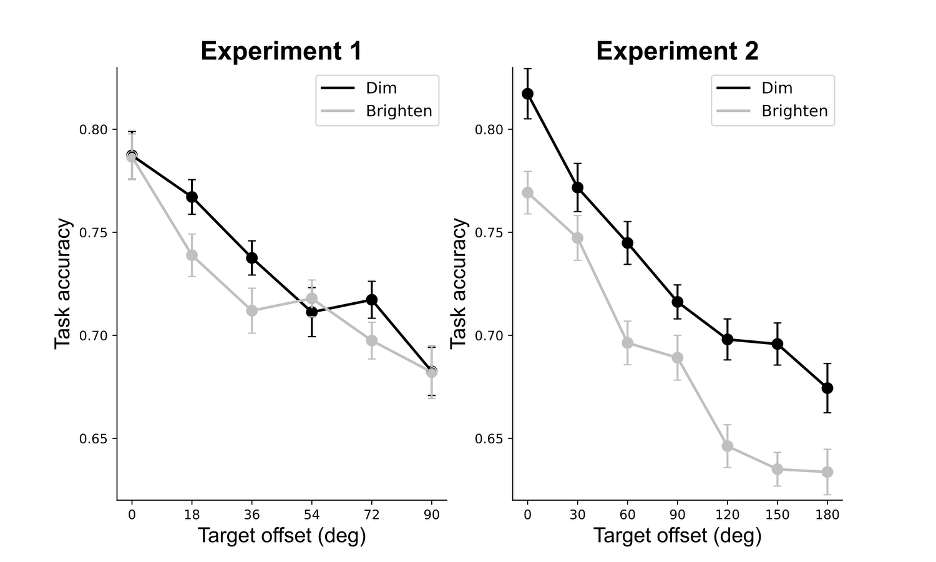


**Figure S3 Results conditioned by response order for Experiment 1 (left panel) and Experiment 2 (right panel)**. The black lines plot accuracies to the first luminance change and the grey lines plot accuracies to the second luminance change. Error bars represent within-subject error as defined by Cousineau (2005).


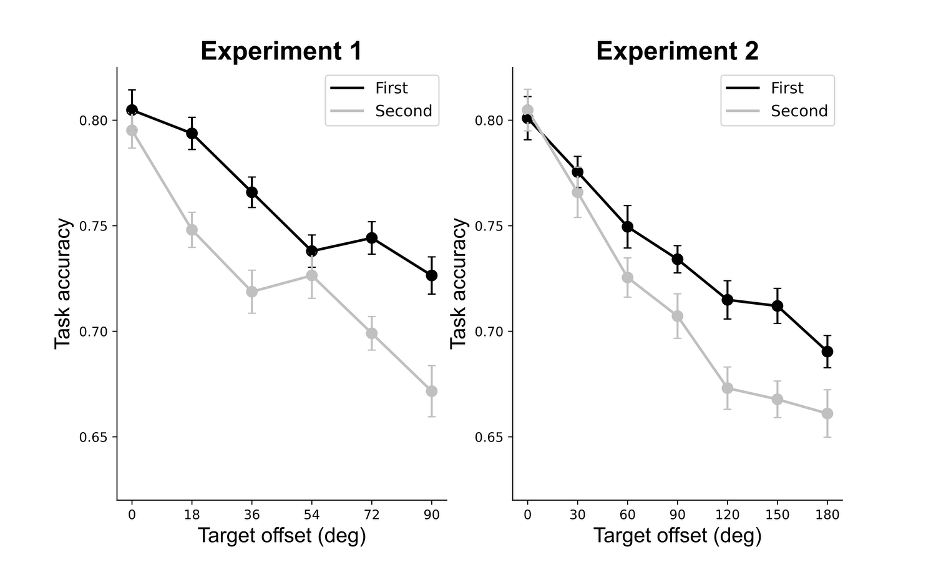


**Figure S4 Results conditioned by quartile of shape wheel for Experiment 1 (left panel) and Experiment 2 (right panel)**. Each of the four lines plot accuracies to shapes within a specific quadrant of the shape wheel shown in Figure 1 which are determined by circular offset on the shape wheel measured in degrees. Error bars represent within-subject error as defined by Cousineau (2005).


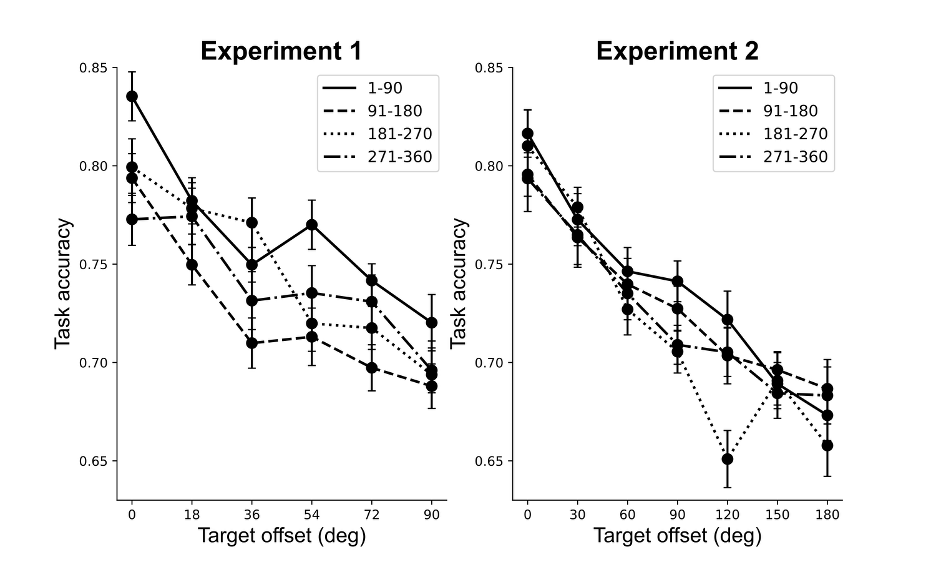


**Figure S5 Results conditioned by target hemifield for Experiment 1 (left panel) and Experiment 2 (right panel)**. The black lines plot accuracies to left hemifield targets while the grey lines plot accuracies to the right hemifield targets. Error bars represent within-subject error as defined by Cousineau (2005).


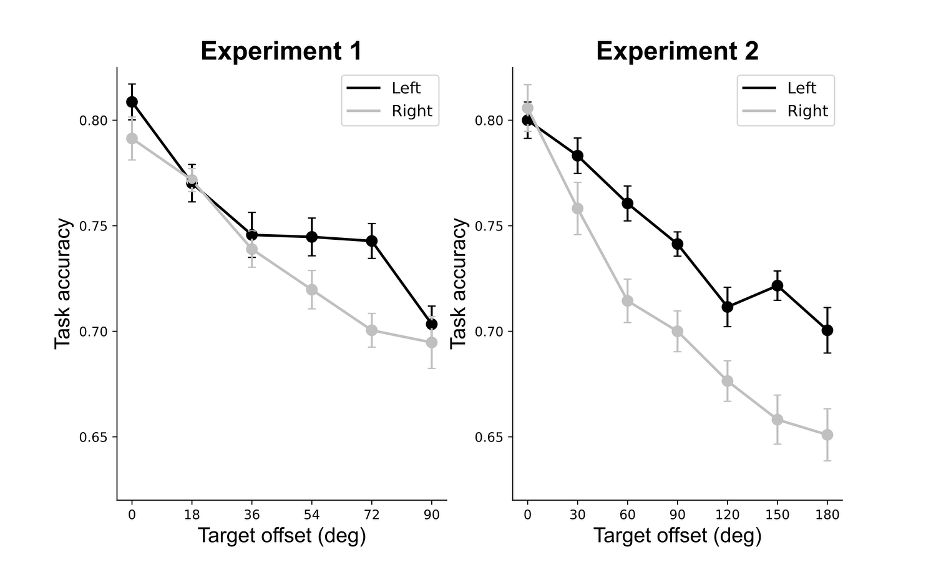


|  | Linear | Gaussian | Exponential |
| --- | --- | --- | --- |
| Experiment 1 | 16 | 8 | 2 |
| Experiment 2 | 13 | 10 | 3 |

**Table S1** The number of individual participants whose data prefer each monotonic model based on Bayesian Information Criteria. The linear model fits the most individual participants’ data for both experiments.

|  | Target Offset Condition | | | | | |
| --- | --- | --- | --- | --- | --- | --- |
|  | 0° | 18° | 36° | 54° | 72° | 90° |
| Hit Rate | .897 (.087) | .879 (.087) | .865 (.089) | .860 (.089) | .850 (.090) | .834 (.089) |
| FA (distractor change) | .196 (.105) | .237 (.105) | .285 (.151) | .309 (.143) | .303 (.148) | .320 (.160) |
| FA (no change) | .091 (.089) | .096 (.111) | .123 (.098) | .111 (.089) | .107 (.086) | .136 (.116) |

**Table S2** The mean hit rates and false alarm rates conditioned by distractors and no change trials for each target offset in Experiment 1. The standard deviation for each mean is given in paratheses. Hit is the proportion of trials in which participants correctly identified a luminance change had occurred for a target, regardless of the sign of the change, which occurred in 60% of trials. The two reported false alarm rates are the proportion of trials in which participants incorrectly reported a target luminance change when a distractor changed (second row), which occurred in 30% of trials or there was no change (third row), which occurred in 10% of trials.

|  | Target Offset Condition | | | | | | |
| --- | --- | --- | --- | --- | --- | --- | --- |
|  | 0° | 30° | 60° | 90° | 120° | 150° | 180° |
| Hit Rate | .891 (.134) | .872 (.134) | .857 (.151) | .834 (.131) | .841 (.121) | .814 (.129) | .796 (.135). |
| FA (distractor change) | .194 (.104) | .249 (.150) | .300 (.167) | .306 (.155) | .365 (.159) | .347 (.179) | .391 (.185) |
| FA (no change) | .098 (.099) | .113 (.120) | .121 (.117) | .105 (.119) | .117 (.099) | .116 (.113) | .132 (.140) |

**Table S3** The mean hit rates and false alarm rates conditioned by distractors and no change trials for each target offset in Experiment 2. The standard deviation for each mean is given in paratheses. Hit is the proportion of trials in which participants correctly identified a luminance change had occurred for a target, regardless of the sign of the change, which occurred in 60% of trials. The two reported false alarm rates are the proportion of trials in which participants incorrectly reported a target luminance change when a distractor changed (second row), which occurred in 30% of trials, or there was no change (third row), which occurred in 10% of trials.
